# Associations of Sex Hormones and Hormonal Status With Arterial Stiffness in a Female Sample From Reproductive Years to Menopause

**DOI:** 10.3389/fendo.2021.765916

**Published:** 2021-11-30

**Authors:** Eija K. Laakkonen, Jari E. Karppinen, Satu Lehti, Earric Lee, Emilia Pesonen, Hanna-Kaarina Juppi, Urho M. Kujala, Eero A. Haapala, Pauliina Aukee, Jari A. Laukkanen, Johanna K. Ihalainen

**Affiliations:** ^1^ Gerontology Research Center, University of Jyväskylä, Jyväskylä, Finland; ^2^ Faculty of Sport and Health Sciences, University of Jyväskylä, Jyväskylä, Finland; ^3^ Institute of Biomedicine, University of Eastern Finland, Kuopio, Finland; ^4^ Department Obstetrics and Gynecology and Pelvic Floor Research and Therapy Unit, Central Finland Health Care District, Jyväskylä, Finland; ^5^ Institute of Clinical Medicine, School of Medicine, University of Eastern Finland, Kuopio, Finland

**Keywords:** vascular aging, vascular stiffness, pulse wave analysis, reproductive hormones, menstruation, hormonal contraception, hormone replacement therapy, women’s health

## Abstract

**Objective:**

Loss of sex hormones has been suggested to underlie menopause-associated increment in cardiovascular risk. We investigated associations of sex hormones with arterial stiffness in 19–58-years-old women. We also studied associations of specific hormonal stages, including natural menstrual cycle, cycle with combined oral contraceptives (COC) and menopausal status with or without hormone therapy (HT), with arterial stiffness.

**Methods:**

This study includes repeated measurements of 65 healthy women representing reproductive (n=16 natural, n=10 COC-users) and menopause (n=5 perimenopausal, n=26 postmenopausal, n=8 HT-users) stages. Arterial stiffness outcomes were aortic pulse wave velocity (PWVao) and augmentation index (AIx%) assessed using Arteriograph-device. Generalized estimating equation models were constructed to investigate associations of each hormone (wide age-range models) or hormonal stage (age-group focused models) with arterial stiffness. PWVao models with cross-sectional approach, were adjusted for age, relative fitness, fat mass and mean arterial pressure, while models with longitudinal approach were adjusted for mean arterial pressure. AIx% models used the same approach for adjustments and were also adjusted for heart rate.

**Results:**

Negative and positive associations with arterial stiffness variables were observed for estradiol and follicle-stimulating hormone, respectively, until adjustment for confounding effect of age. In naturally menstruating women, AIx% was higher at ovulation (B=3.63, p<0.001) compared to the early follicular phase. In COC-users, PWVao was lower during active (B=-0.33 - -0.57, p<0.05) than inactive pills. In menopausal women, HT-users had higher PWVao (B=1.43, p=0.03) than postmenopausal non-HT-users.

**Conclusions:**

When using wide age-range assessments covering reproductive to menopausal lifespan it is difficult to differentiate age- and hormone-mediated associations, because age-mediated influence on arterial stiffness seemed to overrule potential hormone-mediated influences. However, hormonal status associated differentially with arterial stiffness in age-group focused analyses. Thus, the role of sex hormones cannot be excluded. Further research is warranted to resolve potential hormone-mediated mechanisms affecting arterial elasticity.

## Introduction

Cardiovascular (CV) disease is the leading cause of death in postmenopausal women (1). Lack of sex steroid estradiol (E2), either due to natural or surgical menopause, has been found to associate with an increased risk of CV diseases (2–4). Potential mechanisms include both indirect and direct hormone-mediated effects affecting endothelial and smooth muscle cell signaling as well as the extracellular matrix properties of the vasculature (5, 6). Increased arterial stiffness is a clinically important early sign of vascular aging as it predicts future CV events and associates with CV and all-cause mortality (7–9). Increased arterial stiffness refers to a reduced ability of arteries to respond to the pulse wave energy, which can be detected as changes in aortic pulse wave velocity (PWV) and augmentation index (AIx). PWV describes the speed of a pulse wave in the vasculature, while AIx expresses the resistance of the peripheral vascular sites as a reflection of a pulse wave from the peripheral arterial tree (10). The higher the PWV and the AIx are, the stiffer are the arteries. Although PWV increases with age in both men and women, doubling between ages 18 and 80, it is considered to be an indicator of aging-related change in arterial compliance rather than concomitant atherosclerosis (11). Notably, PWV has been found to be lower in women than in aged-matched men throughout reproductive years since puberty until early postmenopause (12), indicating that PWV may be a valuable marker for the arterial stiffening affected by the availability of the sex hormones. AIx, on the other hand, has been shown to be higher in women compared to men throughout adulthood and to adopt a steeper increasing curve at middle-age in both sexes (13).

During a woman’s monthly hormonal cycle, blood concentrations of E2 and other hormones fluctuate considerably according to the menstrual cycle phase. In normal menstrual cycle, E2 levels are the lowest during menstrual bleeding at the early follicular phase and rise thereafter until ovulation. The second E2 peak occurs at the mid luteal phase. A surge of both luteinizing hormone (LH) and follicle-stimulating hormone (FSH) occurs just before ovulation, while progesterone (P4) levels rise after ovulation and are at their highest at the mid luteal phase. In contrast, women with oral contraception (OC) have reduced endogenous hormone production, leading to substantially or completely attenuated peaking of E2, P4, FSH and LH during the menstrual cycle (14). In a study by Hayashi and co-authors, carotid arterial compliance was shown to follow menstrual cycle; compliance increased from early follicular to the ovulatory phase until a sharp decline in the luteal phase (15). However, the study did not find menstrual cycle to associate with peripheral arterial stiffness. In two studies, AIx was shown to follow a similar pattern reaching its peak value during days corresponding to ovulation and dropping thereafter being lowest at the luteal phase (16, 17). The studies did not find AIx to correlate with E2 or P4 (no other hormones were investigated) across menstrual cycle, but the latter study found E2 to correlate negatively with AIx at luteal phase and P4 to correlate negatively with AIx at ovulatory and luteal phase. Other literature reports indicate that the observed changes in AIx or PWV over the natural menstrual cycle are subtle or non-existent (17–21). Only a few studies have investigated differences in arterial stiffness between women with natural menstrual cycle and women using OC and the results have remained inconclusive. One study found PWV to be slightly higher among OC-users than non-users (22), while others found no group differences (23–26). The recent study by Enea et al. (26) compared OC-users at active pill phase to the non-users at bleeding phase and found no difference in PVW but lower AIx in OC-users compared to non-users. This contrasts with findings by Seeland et al., who reported AIx to be higher in OC-users than non-users, accompanied by a negative association with E2 level (25). PVW across OC cycle phases has only been studied by Priest et al. (23) and Yu et al. (24). Neither of the studies found differences in PVW between different pill phases.

After the reproductive stage with cyclic monthly fluctuations in sex hormones, their production in the ovaries gradually declines through perimenopause to postmenopause. This leads to a permanent reduction of circulating E2 and P4 concomitant to heightened FSH levels, which is why menopause is considered to end the protective effects of the hormones towards vasculature (27). Hormone replacement therapy (HT), if started in early menopause, has been suggested to be beneficial for CV health by decreasing both carotid artery intima-media thickness and blood pressure (28), but evidence regarding PWV and AIx have remained inconclusive. The pioneering finding in the field showed PWV to be higher in postmenopausal non-HT-users compared to premenopausal women, and lower in HT-users compared to non-using postmenopausal women (29). However, the later studies have shown mixed results regarding associations of menopausal stage or HT-use with differences in PWV or AIx (30–33). Furthermore, to our knowledge, the association of serum concentrations of sex hormones with both PWV and AIx has not been investigated in menopausal women.

A limited number of studies have investigated arterial stiffness combining both reproductive and menopausal women having either natural hormonal status or taking hormonal preparations. Including hormone assessments into the vascular study models is even rarer. The aim of this study was to investigate the associations of sex hormones with arterial stiffness in a female sample spanning from reproductive years to postmenopause. Furthermore, we investigated the associations of the menstrual cycle, contraception pill cycle and menopausal status with PWV and AIx. We hypothesized that sex hormones influence arterial stiffness, which in our study could be seen as significant negative associations for E2 and P4 values and significant positive associations for FSH and testosterone values with arterial stiffness measurements or between different hormonal states and arterial stiffness.

## Methods

### Participants and Study Design

This study uses data and samples from two studies: the Endogenous and exogenous hormones and performance in women (MEndEx) study and the Estrogen, MicroRNAs and the Risk of Metabolic Dysfunction (EsmiRs) study. MEndEx study protocol has been published in (34). Briefly, healthy women aged 18–40 years were recruited by advertisements in the local newspaper and *via* social media. Each prospective participant was asked to complete a health questionnaire and the Low energy availability in females -questionnaire (LEAF-Q) (35) before the study onset. Inclusion criteria required a participant to be recreationally physically active with a body mass index (BMI) of 18–25 kg/m^2^ and LEAF-Q score < 8. Participants were excluded if they were pregnant or lactating, if they had conditions affecting ovarian function, amenorrhea, endocrine disorders, or chronic diseases, or if they were taking medication that may affect exercise responses. Women using OCs were included. A total of 33 women enrolled for the MEndEx study, of which 28 participated in the measurements. Participated women of reproductive age (REPRO) formed two sub-groups, natural without any type of hormonal contraception (REPRO-NAT group) and combined OC users (REPRO-COC group). Two COC users were excluded from the current study due to lack of arterial stiffness measurement at cycle days of the low estradiol state, i.e., days with withdrawal bleeding during the placebo pill. Finally, 26 young women (16 REPRO-NAT and 10 REPRO-COC) were included in this study. Four experimental testing sessions were completed by each participant over an individual menstrual or contraceptive cycle. The phase of the menstrual or pill cycle in which first experimental testing commenced was randomized. *A priori* familiarization session was scheduled for each participant during which they filled questionnaires and were familiarized to the protocols of the experimental testing sessions. Blood samples were taken, and body composition and vascular measurements were performed in the morning measurements, whereas the cardiorespiratory fitness was assessed later on that same day.

EsmiRs study performed a four-year follow up for the Estrogenic Regulation of Muscle Apoptosis (ERMA) study participants (36). Of the ERMA women, 811 participants who had provided consent to be contacted with new study invitations were approached by postal participation invitation. The EsmiRs study proceeded in three waves; wave-1: postal questionnaire survey, wave-2: laboratory visit repeating ERMA baseline sampling and physiological measurements and wave-3: laboratory visits for metabolic measurements. EsmiRs wave-3 concerned only those women who had participated in wave-2 and did not fulfill additional exclusion criteria. Included participants needed to be either pre-, peri- or postmenopausal, having BMI between 18.5 and 30 kg/m^2^, and being metabolically healthy. E2- and/or P4-containing medication was allowed only for women as menopausal HT. Exclusion criteria were regular use of medication for metabolic disorders (e.g., diabetes, thyroid dysfunction or dyslipidemia), alpha- or beta-blockers, regular use of sedatives or analgesics, and current regular smoking. Occasional smoking was allowed if the participant did not smoke within two days before laboratory visits and not more than a few cigarettes in a month. Wave-3 contained two laboratory visits. The first visit included indirect calorimetry at rest, medical examination and oral glucose tolerance test, and the second visit contained indirect calorimetry during bicycle ergometer test. Vascular measurements were performed at the beginning of both laboratory visits.

The flow of the EsmiRs women to the current study was briefly as follows. Of the 811 invited women, 522 responded (response rate 64.4%), but 28 were not willing to participate. Wave-1 questionnaires were returned by 494 women. Of them, 304 participated in wave-2 laboratory measurements and 42 in wave-3 laboratory measurements. Arterial stiffness measurements were not done for two participants due to the unavailability of the measuring device. One participant turned out to be voluntarily on strict low energy- and carbohydrate diet resulting in ketosis, hence she was excluded from the study. Thus, data was available for the current study from 39 menopausal women (MENO group). Of them, five were perimenopausal without external hormonal preparations (MENO-PERI group), 26 were naturally postmenopausal without the use of HT (MENO-POST group) and eight were postmenopausal women who were currently using HT (MENO-HT group).

The MEndEx and EsmiRs studies adhered to the Declaration of Helsinki. The MEndEx study was approved by the Ethical Committee of the University of Jyväskylä (22 October 2018) and the EsmiRs study by the Ethics committee of the Central Finland Health Care District (9U/2018). All participants received detailed information about the study design, measurements, and procedures and provided signed informed consent before the onset of the study measurements. All laboratory measurements were performed at the Health and Sports Laboratory of the University of Jyväskylä, Finland, during 2019 and 2020.

**Figure 1:**
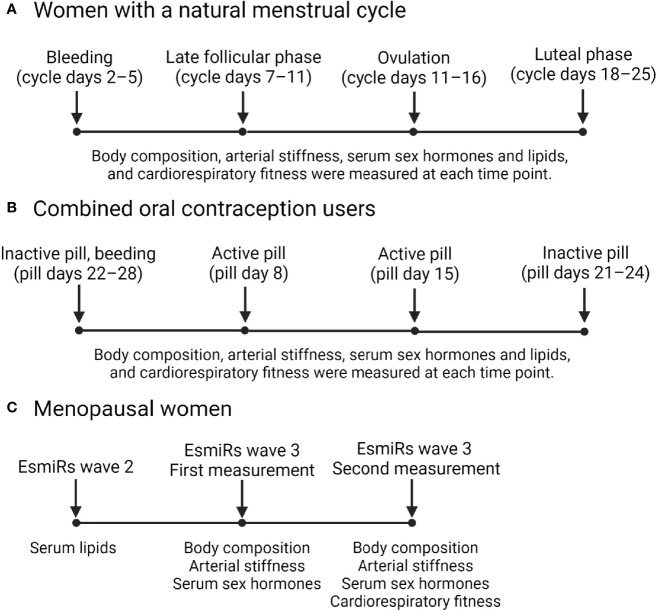
Data collection timepoints in the MEndEx **(A, B)** and EsmiRs **(C)** studies.

**Figure 1** presents a diagram illustrating the timepoints in which data was collected in the MEndEx and EsmiRs. In both studies, the blood samples were drawn on the same morning as the arterial stiffness was assessed.

### Reproductive and Menopausal Stage of the Participants

The REPRO-NAT group included reproductive-age women who did not use any sex hormone containing medications or contraceptives, while the REPRO-COC group included reproductive-age women who were currently using combined estrogen- and progestogen-based OCs. The used products were either third generation COC-pills with ethinyl estradiol coupled with cyproterone acetate (n=2, brand name Vreya) or fourth generation COC-pills with ethinyl estradiol coupled with drospirenone (n=7, brand names Yaz, Yasmin, Stefaminelle and Tasminetta) or estradiol hemihydrate coupled with nomegestrol acetate (n=1, brand name Zoely) as effective hormone components. Active pills of these specimens contain similar amounts of estrogenic (0.02–0.035 mg ethinyl estradiol) and progestogenic (2–3 mg) compounds except that Zoely has 1.5 mg of a bioidentical E2 called estradiol hemihydrate. Taken together, COC-pills used by the MEndEx study participants provide a relatively stable hormonal condition for 21–24 active pill days followed by 4–7 hormone-free (inactive pill) days, although endogenous hormonal profiles may vary between individuals even when OCs employ the same mechanism of action (37).

Menopausal status of the MENO group women was defined according to the Stages of Reproductive Aging Workshop + 10 guidelines (38) using menstrual cycle and serum FSH data. Women who self-reported not have had menstrual bleeding for over 12 months and had at the EsmiRs wave-2 a measured FSH level over 30 IU/L were considered postmenopausal, while women who reported still having occasional menstrual bleedings were considered perimenopausal. Of the postmenopausal women, eight were current HT-users. All MENO-HT group women used bioidentical E2 combined with a synthetic progestogen. Of them, six used an oral product with 1 mg estradiol hemihydrate and 5 mg dydrogesterone (n=5, brand name Femoston-conti) or with 1 mg estradiol valerate and 0.5 mg norethisterone acetate (n=1, brand name Cliovelle), while two used transdermal patches containing either 3.2 mg estradiol hemihydrate and 11.2 mg norethisterone acetate (n = 1, brand name Evorel Conti) or 0.585 mg estradiol hemihydrate with 5 mg oral dydrogesterone (n = 1, brand names Estradot and Terolut).

### Blood Collection and Biochemical Analysis

Fasting venous blood samples were drawn from the antecubital vein at a supine position between 7:00 and 10:00 a.m. From REPRO-NAT women, the samples were drawn at four different data collection weeks representing different menstrual cycle phases. Counting from the onset of menstrual bleeding, samples were drawn on days 2–4 (bleeding), on days 7–11 (follicular phase), on days 11–16 (ovulation), and on days 18–25 (luteal phase) of the menstrual cycle. Ovulation was identified using a combination of counting the days from the commencement of bleeding and daily urine tests starting mid follicular phase (from day 8–9 onwards) to identify the LH surge. Urine was tested by using an at-home ovulation test (Dipro, LH Ovulation Strip, Aidian Oy, Finland). Ovulation occurs within 1–2 days of positive urine test, i.e., LH surge. Thus, ovulation phase laboratory visit was scheduled for 1–2 days after obtaining positive test result and luteal phase laboratory visit was scheduled approximately a week after ovulation (mean 6.3 +/- 0.7 days duration between visits). Women in the REPRO-COC group were also tested at four different timepoints, which are throughout the text presented in the order corresponding to the natural menstrual cycle, i.e., withdrawal bleeding is presented first although it actually occurs at the end of pill cycle after few days of the onset of inactive pill phase. Thus, sampling and measurements which took place at pill days 22–28 were referred as bleeding, at pill day 8 as active pill 1, at pill day 15 as active pill 2 and at pill days 21–24 as inactive pill. Both bleeding and inactive measurement points took place during inactive pill phase. The overlap on days between these phases is due to individual differences on how rapidly withdrawal bleeding starts after changing to the inactive pills. From all MENO women, the blood samples were drawn at wave-2 laboratory visit and at both wave-3 laboratory visits. Wave-2 FSH measurements were used only for the initial group assignments. E2, FSH, P4 and testosterone measurements from both wave-3 sampling points were used in the statistical analyses as week-1 and week-2 timepoints. Lipid and lipoprotein measurements were done only using wave-2 blood samples for the MENO groups while they were available from all sampling weeks for the REPRO-NAT and REPRO-COC groups.

For serum separation, whole blood was left to clot for 30 min at room temperature and centrifuged at 2,200 ×g before aliquoting and storing the sera at −80°C until analysis. Serum LH (only for REPRO groups), E2, P4, FSH and testosterone concentrations were determined using IMMULITE 2000 XPi (Siemens Healthcare Diagnostics, UK) and serum total cholesterol, low-density lipoprotein (LDL)-cholesterol, high-density lipoprotein (HDL)-cholesterol and triglycerides were measured using KONELAB 20 XTi or Indico analyzer (Thermo Fischer Scientific, Finland) according to manufacturer’s instructions.

### Assessment of Aortic Pulse Wave Velocity and Augmentation Index

Participants were instructed to avoid moderate-to-vigorous physical activity 36–48 h before vascular measurements. All measurements were done between 7:00 and 10:00 a.m. Participants rested in a supine position for ten minutes before measurements. Oscillometric pulse wave analysis was then performed from the right upper arm using Arteriograph-device (Arteriograph; TensioMed Ltd., Budapest, Hungary) in the supine position (39). The device provides an automatic assessment of resting heart rate (HR), systolic (SBP) and diastolic blood pressure (DBP), pulse pressure, aortic pulse wave velocity (PWVao), and augmentation index (Aix%). First, the device measures actual systolic blood pressure and subsequently inflates the cuff 35 mmHg above measured SBP, and then measures the fluctuations in the brachial artery. The signals are passed on to a tablet computer, recorded, and analysed as pulse waves. Mean arterial pressure (MAP) was computed by the formula MAP = DBP + 1/3(SBP – DBP). PWVao (m/s) was calculated from the time difference between the first systolic wave (direct) and the second systolic wave (reflected) and was related to the distance from the jugulum to the pubic symphysis. AIx% was computed from the pressure difference between the first (P1) and second (P2) wave in relation to the pulse pressure by the formula AIx% = [(P2-P1)/pulse pressure] x 100. We have previously reported a good short-term reproducibility for PWVao (intraclass correlation coefficient=0.90, coefficient of variation=3.7%) and moderate reproducibility for AIx% (intraclass correlation coefficient=0.88, coefficient of variation=29.1%) in adolescents (40). Within the EsmiRs study sample, using the two repeated measurements that were obtained during data collection wave-3 with a median duration between the measurements being two weeks, moderate reproducibility for PWVao (intraclass correlation coefficient=0.76, coefficient of variation=8.4%) and for AIx% (intraclass correlation coefficient=0.68, coefficient of variation=12.3%) were obtained. Furthermore, Arteriograph-derived PWVao has an acceptable agreement with invasively measured PWVao in adults (41, 42) and with non-invasive tonometric SphymoCor-device (43).

### Anthropometrics and Body Composition

Body weight was measured with a beam scale and height by a stadiometer, with the participant wearing only undergarments. BMI was calculated as weight (kg)/height squared (m^2^). Body composition was assessed with a multifrequency bioelectrical impedance analyzer (InBody™720; Biospace, Seoul, Korea) at the beginning of each laboratory visit in both studies. The InBody-device provides estimates of fat mass and fat-free mass (FFM) of the body and calculates body fat percentage (fat mass (kg)/body weight (kg) x100). It is known that bioimpedance-based methods tend to overestimate FFM, and underestimate fat mass and fat percentage compared to dual-energy x-ray absorptiometry (DXA), which is commonly considered as the golden standard method. However, the use of DXA contains a health risk because each DXA-scan exposes participant to a small amount of radiation. Therefore, its use needs to be carefully justified for repeated measurements. Furthermore, a recent study showed multifrequency bioimpedance to provide similar change estimates of FFM and fat mass compared to the DXA (44). Therefore, we considered InBody ™720 to provide adequate enough estimates of body composition for the purposes of the current study.

### Cardiorespiratory Fitness

In the MEndEx study, peak oxygen uptake (V̇O_2PEAK_) was determined using a standard treadmill test with an incremental protocol at the same time of day +/- 2h to avoid the possible confounding effects of circadian rhythms. An exercise test started with a 5-min warm-up performed at a self-selected pace. The treadmill incline remained constant at 0.5° throughout the entire test. Treadmill velocity was 6 km·h^−1^ for the first 3-min stage of the test and was increased by 1 km·h^−1^ every third minute until volitional exhaustion. HR was recorded continuously using a HR monitor (Polar 800, Polar Electro, Kempele, Finland). The V̇O_2_ was measured breath-by-breath using a portable gas analyzer (Oxycon Mobile^®^, Jaeger, Hoechberg, Germany), which was calibrated before each test according to the manufacturer’s instructions. V̇O_2PEAK_ was defined as the highest average 30 s V̇O_2_ value.

In the EsmiRs study, V̇O_2PEAK_ was determined during an incremental bicycle ergometer test, which consisted of submaximal and maximal phases. All tests were performed between 7:00 and 9:00 a.m. The submaximal phase started at 20 W, and the workload increased for 20 W every four minutes until respiratory exchange ratio (RER) ~1.0 was reached. After that, participants continued directly to the maximal phase. The maximal phase started at 100 W, and the workload was increased 1 W/3 s (20 W/min) until voluntary exhaustion. Participants were instructed to cycle the ergometer (Ergoselect 200, Ergoline GmbH, Germany) at 70 ± 5 rpm. HR and workload were recorded continuously with a 12-lead ECG -system (CardioSoft v.5.02, GE Medical System Corina, GE Medical System Inc., USA). Gas exchange was measured with Vmax Encore 92 metabolic cart (Sensormedics, Yorba Linda, CA, USA), which was calibrated before each test according to the manufacturer’s instructions. Gas exchange data were averaged for every 10 s, and V̇O_2PEAK_ was determined as the highest rolling 30 s of V̇O_2._ We used cutoffs RER_MAX_ ≥ 1.10 or age-predicted maximal heart rate (210 - age) ≥ 99% to assess whether exercise test was maximal. One participant did not quite meet the set limits, but her data was still considered reliable. Respiratory gas exchange data was unreliable in two participants due to equipment failure or difficulties wearing the mask. For these two participants, V̇O_2PEAK_ was determined from W_MAX_ using the equation by Storer et al. (45).

For all study participants, the relative V̇O_2PEAK_ was computed as V̇O_2PEAK_ relative to FFM (V̇O_2PEAK_/FMM) obtained by bioimpedance measurements and expressed as ml/kg FFM/min.

### Statistics

Means, standard deviations and frequencies were used for descriptive purposes. When available, data points which are commonly considered to represent the lowest E2 phase of the menstrual or pill cycle, i.e., menstrual bleeding at early follicular stage and withdrawal bleeding during placebo pill, were used to describe REPRO-NAT and REPRO-COC groups and as references in generalized estimating equation (GEE) models. Correspondingly, for the MENO group, MENO-POST group was chosen as a reference in the GEE models. In descriptive tables, the MENO group week-1 data point was used except for one participant whose week-2 data was used due to missing data at week-1. Group comparisons between REPRO and MENO groups and between REPRO-NAT and REPRO-COC groups were performed using Student’s t-test or, if Shapiro-Wilk test indicated variable not to follow a normal distribution, Mann-Whitney U test. Group comparisons between MENO-PERI, -POST and -HT groups were correspondingly done using ANOVA or Kruskall-Wallis test. Linear GEE models with an unstructured working correlation matrix were constructed for the complete dataset to investigate potential associations of hormones (wide age-range models) with PWVao and AIx%. Age-group focused GEE models were also constructed for each group to investigate associations of menstrual cycle phases (REPRO-NAT group, four datapoints), COC-pill cycle phases (REPRO-COC group, four datapoints) and menopausal stages (two datapoints for each MENO group) with PWVao and AIx%. The models with cross-sectional nature (wide age-range models and of the age-group focused models, the MENO group model) were adjusted for age, relative cardiorespiratory fitness level (V̇O_2PEAK_/FMM), body fat mass, MAP and AIx% models also for heart rate to avoid differences in these metrics to influence the interpretation of the results. The models with longitudinal nature (of the age-group focused models, REPRO-NAT and REPRO-COC group models) were adjusted only for MAP, and in the case of AIx%, also for heart rate. Regression coefficients (B), standard errors (SE), *P*-values and 95% confidence intervals (95% CI) are reported for each model, and both unadjusted and adjusted models are presented. The benefit of GEE model over repeated-measures ANOVA is that participants are not lost due to incomplete observations (e.g., from participants who have information available from only some of the datapoints) (46). In the REPRO-NAT group, there was missing data for PWVao and AIx% for one woman at follicular phase, three women at ovulation phase and two women at luteal phase, for V̇O_2PEAK_/FMM for two women at bleeding phase, two women at follicular phase, four women at ovulation phase and for two women at luteal phase and for E_2_, FSH, P4 and testosterone for two women at ovulation phase. In the REPRO-COC group, there was missing data for PWVao and AIx% for one woman at inactive pill phase, and for V̇O_2PEAK_/FMM for two women at bleeding phase and one woman at active pill 1 phase. In the MENO group, there was missing data for PWVao and AIx% for one woman at week-1 and two women at week-2. *P* ≤ 0.05 was considered statistically significant. All statistical analyses were performed using IBM SPSS Statistics 24.0.

## Results

### Descriptive Characteristics and Hormonal Status of the Study Participants

**Table 1** presents descriptive characteristics of the study participants. Participated women were 26 adult 19 to 37 years old women representing the reproductive-age period and 39 adult 52 to 58 years old women representing the menopause-age period. All women in the REPRO group and 74% of the MENO group women were never smokers. Of the MENO women, five were perimenopausal and 34 were postmenopausal. Of the REPRO group, 62% and of the MENO group, 80% had natural status without the current use of external hormone preparations. All participants combined, ten were current COC-users, nine had previously used progestogen-releasing coils while eight were current and one was a former HT-user.

**Table 1:** Descriptive characteristics of the study participants.

	REPRO group	MENO group
	*n* = 26	*n* = 39
Age (years)	24.9 ± 4.2	55.0 ± 1.7
Smoking, *n* (%)		
Never	26 (100)	29 (74.4)
Former		8 (20.5)
Occasional		2 (5.1)
Current reproductive status, *n* (%)		
Reproductive, regularly menstruating, natural	16 (61.5)	–
Reproductive, regularly menstruating, COC-user	10 (38.5)	–
Perimenopausal, irregular cycle	–	5 (12.8)
Postmenopausal, none menstruating, natural	–	26 (66.7)
Postmenopausal, none menstruating, HT-user	–	8 (20.5)
Use of hormonal contraceptives, *n* (%)		
Never	16 (61.5)	30 (77.9)
Former per type		
IUD, hormonal coil	–	9 (23.1)
Current per type		
Oral combined	10 (38.5)	–
Use of menopausal hormonal therapy, *n* (%)		
Never	26 (100)	31 (77.5)
Former		1 (2.5)
Current per type		
Oral combined		6 (15.0)
Transdermal combined		2 (5.0)
Estradiol (pmol/l)	249.8 ± 147.8	181.8 ± 204.1
Follicle-stimulating hormone (IU/l)	4.9 ± 2.5	71.4 ± 36.0
Progesterone (nmol/)	1.6 ± 1.4	1.1 ± 4.5
Luteinizing hormone (IU/l)	4.8 ± 3.4	–
Testosterone (nmol/l)	0.8 ± 0.6	0.5 ± 0.3

Values are mean ± standard deviation unless otherwise stated. COC, combined oral contraception; HT, hormone therapy; IUD, intrauterine device.

**Figure 2:**
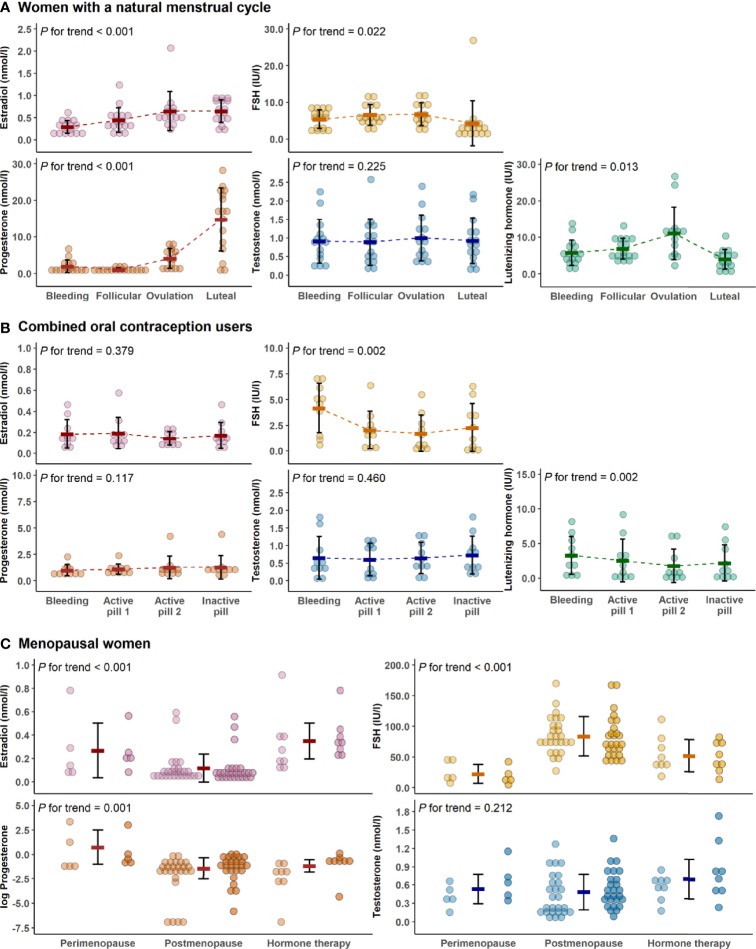
Hormone levels across data collection time points and study groups. Hormone concentrations during the menstrual cycle of the natural reproductive women **(A)**, during pill cycle of the combined oral contraceptive users **(B)** and among menopausal women **(C)**. In all figures, bold lines in graphs represent means with standard deviations. For menopausal women, the means were calculated as average concentrations of the two measurement points (the first measurement point is presented with lighter and the second is with darker colors). In C, progesterone was log-transformed for better visualization purposes only. P for trend is provided by Wald Chi-Squared Test of the generalized estimating equation models. FSH, follicle-stimulating hormone.

**Figure 2** presents the variation of hormone levels between different menstrual and COC-pill cycle phases and menopausal groups. The typical menstrual cycle dependent variation is evident for E2, P4, LH, and FSH, while testosterone did not change at group mean level among the naturally menstruating women (REPRO-NAT group, **Figure 2A**). In the REPRO-COC group, there were only modest differences in LH and FSH levels across the pill cycle phases with active and inactive pills (**Figure 2B**). For the menopausal women, statistics were performed as means of the two data collection weeks and compared between MENO-PERI, -POST and -HT groups (**Figure 2C**). As expected, E2 levels were lowest and FSH levels were highest among MENO-POST women. Considerable variation was observed in P4 among MENO-PERI women indicating that participants still had an active menstrual cycle.

### Indicators of Cardiovascular Health of the Study Participants

**Figure 3:**
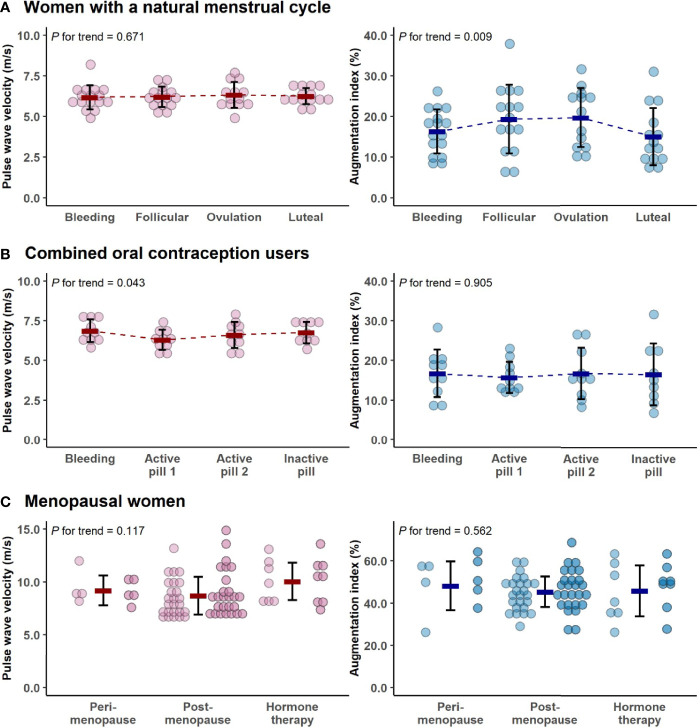
The variations in pulse wave velocity and augmentation index within the menstrual cycle **(A)**, combined oral contraceptive-pill cycle phases **(B)** and menopausal groups **(C)**. In all figures, bold lines represent means with standard deviations. For menopausal women, the means were calculated as average concentrations of the two measurement points (the first measurement point is presented with lighter and the second is with darker colors). Note the differing y-axes in younger and older women. P for trend is provided by Wald Chi-Squared Test of the generalized estimating equation models.

Most of the measured parameters indicated REPRO group to have better CV health than menopausal women (**Table 2**). REPRO group had higher absolute and relative V̇O_2PEAK_ than MENO group. They also had healthier body composition as indicated by lower BMI and fat mass and higher FFM. Total cholesterol and LDL-cholesterol levels were lower in the REPRO than in the MENO group. REPRO group also had lower SBP, DBP, PWVao and AIx% values indicating less rigid arteries compared to the MENO group. For the sub-group mean values and comparisons between REPRO-NAT to REPRO-COC and between MENO-PERI, -POST and -HT groups see **Table S1**. Of the women of reproductive age, REPRO-COC women had an average higher triglyceride, BMI, SBP, DBP and PWVao values than REPRO-NAT women. No within-group differences among menopausal women were observed. The variations in PWVao and AIx% within the menstrual cycle and COC-pill cycle phases are presented in **Figure 3**.

**Table 2:** Parameters of cardiovascular health.

	REPRO group	MENO group	*P*-value
	*n* = 26	*n* = 39	
**Blood lipids and lipoproteins** ^a^			
Triglycerides (mmol/l)	0.8 ± 0.3	1.0 ± 0.4	0.065
Total cholesterol (mmol/l)	4.6 ± 0.8	5.8 ± 1.1	**<0.001^c^**
LDL-cholesterol (mmol/l)	2.4 ± 0.6	3.5 ± 1.0	**<0.001^c^**
HDL-cholesterol (mmol/l)	1.9 ± 0.3	2.0 ± 0.5	0.131
**Body composition**			
Body mass index (kg/m^2^)	22.9 ± 2.6	24.5 ± 2.6	**0.018**
Fat free mass (kg)	51.6 ± 5.9	46.4 ± 4.7	**<0.001**
Fat mass (kg)	13.4 ± 4.7	21.2 ± 5.3	**<0.001**
**Cardiorespiratory fitness** ^b^			
V̇O_2PEAK_ (l/min)	2.9 ± 0.4	2.1 ± 0.4	**<0.001**
Relative VO_2PEAK_ (ml/kg FFM/min)	57.1 ± 9.5	45.9 ± 6.3	**<0.001^c^**
Resting heart rate (bpm)	58.2 ± 11.1	57.1 ± 8.5	0.835 **^c^**
**Blood pressure and arterial stiffness**			
Systolic blood pressure (mmHg)	111.8 ± 9.8	124.0 ± 15.1	**0.001^c^**
Diastolic blood pressure (mmHg)	63.0 ± 8.5	72.7 ± 7.7	**<0.001**
Mean arterial pressure (mmHg)	79.3 ± 8.5	89.8 ± 9.7	**<0.001**
Aortic pulse wave velocity (m/s)	6.4 ± 0.8	9.1 ± 1.9	**<0.001^c^**
Augmentation index (%)	16.5 ± 5.5	45.9 ± 10.1	**<0.001^c^**

Values are mean ± standard deviation. V̇O_2PEAK_, peak oxygen uptake, FFM, fat free mass, LDL, low-density lipoprotein, HDL, high-density lipoprotein. Missing data: ^a^REPRO group: n=1, ^b^REPRO group: n=4. ^c^Mann-Whitney U test was used for statistical testing. Significant P-values are bolded.

### Association of Sex Hormones With Arterial Stiffness

Age is known to associate with arterial stiffness, which was also the case in our sample; age association with PWVao was B= 0.09, 95% CI 0.07 to 0.11 (p < 0.001) and with AIx% B= 0.95, 95% CI 0.84 to 1.06 (p < 0.001). The associations of the measured hormones with PWVao and AIx% were investigated with GEE-models presented in **Tables 3**, **4**, respectively. Associations were first inspected using univariable models, which were then adjusted for age (multivariable model 1) and finally for age and relative fitness level, body fat mass, MAP, and for AIx% also for heart rate (multivariable model 2). E2 was not associated with AIx% in any of the models but it was negatively associated with PWVao in the univariable model (B=-0.55, p=0.05). However, adjustment for age attenuated the association between E2 and PWVao. Statistically significant association between age and PWVao remained even after further adjustments (multivariable model 2). FSH was positively associated with PWVao and AIx% in unadjusted models (B=0.04, p=0.038 and B=0.26, p<0.001, respectively). Further adjustment for age attenuated these associations. Age remained to be significantly associated with AIx% but not with PWVao in FSH multivariable model 2. P4 was not associated with PWVao or AIx% in univariable models and regarding AIx% in multivariable models. However, regarding PWVao, the P4 association became statistically significant after adjustment for age (multivariable model 1: B=-0.16, p=0.025) but disappeared when other covariates were added into the model (multivariable model 2). Testosterone was not associated with PWVao or AIx% in any of the GEE models. Age was positively associated with both PWVao and AIx% in P4 and testosterone multivariable models.

**Table 3:** Associations of estradiol, follicle-stimulating hormone, progesterone, and testosterone with aortic pulse wave velocity.

PWVao	Estradiol models [nmol/l]		FSH models [IU/l]		Progesterone models [nmol/l]		Testosterone models [nmol/l]	
	B (SE)	95% CI	B (SE)	95% CI	B (SE)	95% CI	B (SE)	95% CI
**Univariable model**								
Hormone	-0.55 (0.28)*	-1.11 to 0.01	0.04 (0.02)*	0.01 to 0.08	-0.01 (0.01)	-0.02 to 0.01	-0.23 (0.17)	-0.57 to 0.10
**Multivariable model 1**								
Hormone	-0.27 (0.33)	-0.92 to 0.39	0.04 (0.03)	-0.01 to 0.09	-0.01 (0.01)	-0.02 to 0.02	-0.06 (0.16)	-0.39 to 0.27
Age	0.09 (0.01)***	0.07 to 0.11	0.04 (0.06)	-0.07 to 0.14	0.09 (0.01)***	0.07 to 0.11	0.09 (0.01)***	0.07 to 0.11
**Multivariable model 2**								
Hormone	-0.01 (0.22)	-0.44 to 0.42	0.02 (0.01)	-0.01 to 0.04	0.01 (0.02)	-0.03 to 0.05	-0.01 (0.24)	-0.46 to 0.47
Age	0.08 (0.02)***	0.04 to 0.13	0.04 (0.04)	-0.03 to 0.12	0.09 (0.02)***	0.04 to 0.13	0.09 (0.02)***	0.04 to 0.13
V̇O_2PEAK_/FFM	-0.05 (0.04)	-0.13 to 0.02	-0.05 (0.03)	-0.12 to 0.01	-0.05 (0.03)	-0.12 to 0.01	-0.05 (0.04)	-0.12 to 0.02
Fat mass	-0.13 (0.06)*	-0.24 to -0.01	-0.11 (0.06)*	-0.22 to -0.01	-0.13 (0.06)*	-0.25 to -0.01	-0.13 (0.06)*	-0.25 to -0.02
MAP	0.06 (0.02)**	0.02 to 0.10	0.05 (0.02)**	0.02 to 0.09	0.06 (0.02)**	0.02 to 0.10	0.06 (0.02)**	0.02 to 0.10

*P ≤ 0.05, **P ≤ 0.01, ***P ≤ 0.001. SE, standard error; CI, confidence interval; V̇O_2PEAK_, peak oxygen uptake; FFM, fat free mass; MAP, mean arterial pressure; FSH, follicle-stimulating hormone.

**Table 4:** Associations of estradiol, follicle-stimulating hormone, progesterone, and testosterone with augmentation index.

AIx%	Estradiol models [nmol/l]		FSH models [IU/l]		Progesterone models [nmol/l]		Testosterone models [nmol/l]	
	B (SE)	95% CI	B (SE)	95% CI	B (SE)	95% CI	B (SE)	95% CI
**Univariable model**								
Hormone	-1.69 (2.61)	-6.80 to 3.42	0.26 (0.03)***	0.21 to 0.31	-0.08 (0.11)	-0.30 to 0.15	2.35 (3.41)	-4.34 to 9.03
**Multivariable model 1**								
Hormone	-2.26 (1.45)	-5.10 to 0.58	0.03 (0.04)	-0.04 to 0.11	-0.16 (0.07)*	-0.30 to -0.02	-0.24 (1.36)	-2.89 to 2.42
Age	0.94 (0.05)***	0.83 to 1.05	0.89 (0.11)***	0.68 to 1.09	0.94 (0.05)***	0.83 to 1.05	0.94 (0.05)***	0.84 to 1.05
**Multivariable model 2**								
Hormone	-2.29 (2.13)	-6.47 to 1.89	0.02 (0.03)	-0.04 to 0.08	-0.07 (0.06)	-0.18 to 0.05	0.02 (1.30)	-2.53 to 2.53
Age	0.89 (0.07)***	0.75 to 1.03	0.82 (0.10)***	0.62 to 1.02	0.86 (0.07)***	0.71 to 1.00	0.85 (0.08)***	0.70 to 1.00
V̇O_2PEAK_/FFM	0.15 (0.12)	-0.08 to 0.38	0.17 (0.13)	-0.08 to 0.42	0.11 (0.13)	-0.14 to 0.35	0.19 (0.14)	-0.08 to 0.47
Fat mass	-0.09 (0.16)	-0.41 to 0.23	-0.03 (0.17)	-0.36 to 0.31	-0.03 (0.18)	-0.38 to 0.33	-0.01 (0.17)	-0.35 to 0.33
MAP	0.35 (0.06) ***	0.22 to 0.47	0.37 (0.07) ***	0.24 to 0.51	0.37 (0.06) ***	0.25 to 0.49	0.39 (0.08)***	0.25 to 0.55
HR	-0.34 (0.07)***	-0.48 to -0.20	-0.36 (0.08)***	-0.52 to -0.20	-0.34 (0.08)***	-0.50 to -0.18	-0.36 (0.09)***	-0.54 to -0.18

*P ≤ 0.05; ***P ≤ 0.001. SE, standard error; CI, confidence interval; V̇O_2PEAK_, peak oxygen uptake; FFM, fat free mass; MAP, mean arterial pressure; HR, resting heart rate; FSH, follicle-stimulating hormone.

### Association of the Menstrual Cycle, Contraceptive Pill Cycle and Menopausal Status With Arterial Stiffness

Associations of the menstrual cycle phases with arterial stiffness were investigated within the REPRO-NAT group (**Table 5**). The menstrual cycle phase was associated with AIx% but not with PWVao. AIx% was higher during follicular and ovulation phases compared to the bleeding phase (**Figure 3**) but in the GEE model adjusted for MAP and heart rate, only ovulation phase (B=3.04, p=0.003) differed statistically significantly from the bleeding phase (**Table 5**).

**Table 5:** Association of menstrual cycle stages with arterial stiffness.

	PWVao models [m/s]		AIx% models [%]	
	B (SE)	95% CI	B (SE)	95% CI
**Univariable model**				
Bleeding (ref)				
Follicular	0.02 (0.12)	-0.22 to 0.26	2.63 (1.35)*	-0.02 to 5.28
Ovulation	0.10 (0.11)	-0.11 to 0.31	2.84 (1.09)**	0.71 to 4.97
Luteal	0.10 (0.14)	-0.17 to 0.37	-0.61 (1.06)	-2.68 to 1.46
**Multivariable model**				
Bleeding (ref)				
Follicular	-0.02 (0.14)	-0.30 to 0.26	2.53 (1.38)	-0.17 to 5.25
Ovulation	0.10 (0.12)	-0.14 to 0.34	3.04 (1.04)**	1.01 to 5.07
Luteal	0.02 (0.15)	-0.27 to 0.31	-0.77 (1.02)	-2.76 to 1.22
MAP	0.04 (0.02)***	0.03 to 0.09	0.25 (0.15)	-0.05 to 0.55
HR			-0.08 (0.11)	-0.30 to 0.13

*P ≤ 0.05, **P ≤ 0.01, ***P ≤ 0.001. PWVao, aortic pulse wave velocity, AIx%, augmentation index; SE, standard error; CI, confidence interval; ref, reference group; MAP, mean arterial pressure; HR, resting heart rate.

Associations of the contraceptive pill cycle with arterial stiffness were investigated within the REPRO-COC group (**Table 6**). The pill cycle was associated with PWVao but not with AIx%. Our data indicates that PWVao may be at a slightly lower level during the first week of active pill than other weeks (**Figure 3**). In the MAP adjusted GEE model, PWVao was lower during the first (B=-0.46, p<0.001), but not during the second (B=-0.17, p=0.371) active pill week compared to the withdrawal bleeding days during the placebo pill.

**Table 6:** Association of pill cycle stages with arterial stiffness.

	PWVao models [m/s]		AIx% models [%]	
	B (SE)	95% CI	B (SE)	95% CI
**Univariable model**				
Bleeding (ref)				
Active pill 1	-0.57 (0.21)*	-0.98 to -0.17	-1.01 (2.07)	-5.06 to 3.04
Active pill 2	-0.26 (0.16)	-0.57 to 0.05	-0.5x10^-15^ (1.37)	-2.68 to 2.68
Inactive pill	-0.12 (0.10)	-0.31 to 0.08	-0.31 (1.36)	-2.98 to 2.37
**Multivariable model**				
Bleeding (ref)				
Active pill 1	-0.46 (0.13)***	-0.71 to -0.21	-0.61 (1.69)	-3.93 to 2.72
Active pill 2	-0.17 (0.19)	-0.55 to 0.20	-1.47 (1.21)	-3.84 to 0.90
Inactive pill	-0.08 (0.12)	-0.32 to 0.15	0.12 (0.95)	-1.74 to 1.97
MAP	0.25 (0.03)	-0.03 to 0.08	0.44 (0.06) ***	0.31 to 0.56
HR			-0.44 (0.08)***	-0.60 to -0.28

*P ≤ 0.05, ***P ≤ 0.001. PWVao, aortic pulse wave velocity; AIx%, augmentation index; SE, standard error; CI, confidence interval; ref, reference group; MAP, mean arterial pressure; HR, resting heart rate.

Associations of the menopausal stage with arterial stiffness were investigated within the MENO group (**Table 7**). Menopausal status was not associated with AIx%. Postmenopausal HT-users had higher PWVao (B=2.00, p<0.001) compared to the MENO-POST group in the multivariable model adjusted for age, relative fitness level, body fat mass and MAP.

**Table 7:** Association of menopausal stages with arterial stiffness.

	PWVao models [m/s]		AIx% models [%]	
	B (SE)	95% CI	B (SE)	95% CI
**Univariable model**				
MENO-POST (ref)				
MENO-PERI	0.53 (0.64)	-0.72 to 1.77	5.29 (5.05)	-4.61 to 15.18
MENO-HT	1.38 (0.67)*	0.07 to 2.70	1.36 (4.18)	-6.84 to 9.56
** Multivariable model**				
MENO-POST (ref)				
MENO-PERI	0.32 (0.80)	-1.24 to 1.88	0.91 (2.87)	-4.71 to 6.54
MENO-HT	2.00 (0.43)***	1.16 to 2.84	4.67 (2.49)	-0.20 to 9.55
Age	-0.01 (0.16)	-0.32 to 0.31	0.90 (0.62)	-0.31 to 2.10
V̇O_2PEAK_/FMM	0.01 (0.04)	-0.07 to 0.09	-0.01 (0.13)	-0.27 to 0.27
Fat mass	-0.11 (0.05)*	-0.21 to -0.01	-0.57 (0.14)***	-0.84 to -0.29
MAP	0.12 (0.04)***	0.05 to 0.19	0.66 (0.07)***	0.52 to 0.81
HR			-0.70 (0.08)***	-0.85 to -0.56

*P ≤ 0.05, ***P ≤ 0.001. SE, standard error; CI, confidence interval; MENO-POST, postmenopausal women with natural status; MENO-PERI, perimenopausal women with natural status; MENO-HT, postmenopausal hormone therapy users; ref, reference group, V̇O_2PEAK_, peak oxygen uptake; FFM, fat free mass; MAP, mean arterial pressure; HR, resting heart rate.

## Discussion

We investigated the potential involvement of sex hormones in arterial stiffening during women’s lifespan from reproductive to postmenopausal years. In our whole study sample, the measured hormones showed weak negative (E2), weak positive (FSH) or non-existent (P4 and testosterone) association with PWVao. Regarding AIx%, a significant positive association was found only with FSH. However, these associations were largely explained by the confounding effect of age. We also studied arterial stiffness within each age-group characterized by differing sex hormone profiles. Among women of reproductive age, those women who had natural menstrual cycle had higher AIx% at ovulation phase compared to bleeding, while among COC-users, PWVao was lower in the active pill phase (week 1) compared to the inactive pill phase with withdrawal bleeding. Among menopausal women, HT-user had higher PWVao than postmenopausal non-HT-users. Finding significant associations with arterial stiffness variables in age-group focused models indicates that differing sex hormone profiles may influence elastic properties of the arteries.

### Are Sex Hormones Driving Vascular Stiffening?

Large population studies have shown arterial stiffness to associate with heart failure risk (47) and with the risk of CV diseases (48, 49). Furthermore, the meta-analysis by Vlachopoulos et al. (7) showed that PVW predicts increased risk of CV events and mortality. Since the menopausal loss of E2 is known to associate with CV disease (2–4), it is important to understand if E2 or other sex hormones may have an influence on arterial stiffness. Our results indicated that sex hormones, especially E2 and FSH, may associate with arterial stiffness. However, the more detailed investigations revealed that hormone associations were largely explained by age. Therefore, our study strengthens the evidence that age is an important variable when explaining women’s arterial stiffness in wide age-range studies. Hormones may function as mediators of the arterial stiffening process; however, the mechanisms should be further studied to draw any definitive conclusions. It is also possible that the associations between hormones and arterial stiffness are different at different ages or different reproductive phases of a woman’s life. At least in our hands, when the study sample had a wide age range, adding direct hormone measurements to the analysis did not provide additional value.

Even though age explained arterial stiffness in the whole study population, our age-group focused analyses indicated that hormonal status may still have a role in arterial elasticity regulation. This is notable, as among young women in reproductive age, the use of oral contraceptives for birth control is common (50). Third and fourth generation COC-pills work by blunting the natural monthly hormonal cycle, as also seen in this study (**Figure 2**). Therefore, it is important to understand if natural hormone fluctuations or their absence affect arterial stiffness. Among reproductive women with natural menstrual cycles, we observed higher AIx% at ovulation compared to the early follicular phase, while among COC-users, PWVao was lower during the active pill than in the inactive pill phase. Our results on menstrual cycle association with AIx% are supported by similar results obtained by Robb and co-workers (16) and by Umapathy and co-workers (17); however, one other study found an association to the inverse direction (20). Another study (19) did not find menstrual cycle to associate with AIx%, but the researchers did not include the ovulatory phase in their study. None of the studies thus far, including ours, have found the menstrual cycle to associate with PVW (17–20). We have not found other reports showing differential associations on COC-pill cycle phases with PVW. Thus far, studies have reported no differences in PVW across COC-pill phases (23, 24).

The explanation for the seemingly directionally opposite association of menstrual and COC-pill cycles with arterial stiffness may lie in systemic hormonal status differences at the selected reference phases. In women with a natural menstrual cycle, other cycle phases were compared to the early follicular bleeding phase, when all sex hormones reached their lowest values, and the difference was seen in comparison to the phase with elevated E2, FSH, P4 and peaking LH. Conversely, the comparisons in COC-users were performed between the hormonal suppression phase of endogenous hormones with concurrent small pill dosage-dependent daily peaking of synthetic forms of estrogen and progestogen (active pill) and the wash-out period with withdrawal bleeding (inactive pill). In our study, most COC-users used either third or fourth generation COC-pills containing ethinyl estradiol coupled with cyproterone or drospirenone, and one woman used fourth generation COC-pills containing estradiol hemihydrate coupled with nomegestrol acetate. Unfortunately, we were not able to measure systemic levels of these exogenous hormones, which may also affect arteries (51, 52). The only endogenous hormones showing pill-cycle differences among COC-users were FSH and LH, both being slightly higher during our reference phase with inactive compared to active pill weeks. This is the opposite of the natural menstrual cycle when FSH and LH but also E2 and P4 levels rise towards ovulation and are at their lowest during menstrual bleeding. Therefore, it is plausible to consider FSH and less likely also LH to be the hormonal driving forces associated with vascular stiffness among reproductive-age women. However, we do not mean to reject the potential role of exogenous hormones, especially among COC- and HT-users, from the hypothesis. Different exogenous hormone combinations used in COC-pills have been found to affect for instance endothelium-dependent vasodilatation (51, 52), while some conflicting results have been obtained regarding exogenous hormones released due to the use of HT-pills. Use of HT-pills comprised of 17β-estradiol and gestodene was shown not to affect arterial distensibility (53), while transdermal HT with 17β-estradiol and norethisterone acetate was shown to improve arterial compliance (54). To our knowledge, there are no studies investigating the potential role of LH in arterial stiffening at any hormonal stage of a woman’s life. FSH and other sex hormones have also been rarely directly studied with arterial stiffness measures. Hildreth et al. (2013) reported postmenopausal women’s FSH level to correlate negatively with carotid artery compliance, while E2, P4 and estrone correlated positively (55). Current literature regarding PWV and postmenopausal HT use has remained inconclusive and has not investigated direct associations with systemic hormone levels before our study. Some studies have shown aortic stiffness to be lower among HT-users compared to postmenopausal non-users (29, 56), but others have found no difference (30, 31). Contrarily, we found PWVao to be higher among HT-users than non-users.

Inconsistencies also remain in hormone-measuring studies with women of reproductive age. Similar to our results, Robb et al. (2009) reported AIx to be at its highest range during ovulation, but they did not found correlation between E2 or P4 with AIx (16). Other sex hormones or PWV were not studied. Based on our literature searches, the only previous study, which has investigated associations between systemic endogenous hormone levels and arterial stiffness among OC-users, was done by Seeland and co-workers (2020) (25). They found AIx, but not PWV, to be higher in OC-users compared to the non-users, and AIx to be negatively associated with E2 concentration. However, they did not investigate differences in arterial stiffness during different menstrual or pill cycles, nor did they report at which cycle phase the measurements were performed. As far as we know, a study by Priest et al. (2018) (23) is the only one that has investigated the potential effect of menstrual or pill cycle as well as sex hormones (E2, P4 or testosterone) on arterial stiffness and found none. In our study, PWVao was slightly higher among reproductive COC-users than non-users, and active pill cycle phases were negatively associated with PWVao, while no differences were observed for AIx%. Furthermore, we found PWVao, not AIx%, to be negatively associated with E2 in the wide age-range analyses. More research is needed to resolve potential direct hormonal effects on arterial stiffening over the menstrual cycle, OC-pill cycle, and menopausal transition. In this sense, FSH rises as a potential new point of interest alongside other sex hormones. OC-mediated suppression of endogenous hormones and inclusion of exogenous hormones also deserves to be investigated more thoroughly. The current knowledge base is incomplete due to non-reporting or non-investigating menstrual and OC-pill cycle phases and due to variegated use and reporting practices of hormonal medications.

It is important to note that hormonal state and ovarian aging may also influence women’s arterial functions and CV health through indirect routes such as lipid and glucose metabolism, blood pressure and body composition (56). A recent publication from a multicenter randomized clinical trial showed that higher E2 dose received by early postmenopausal women led to lower triglyceride and LDL-cholesterol and higher HDL-cholesterol levels while no effect due to P4 was observed (57). Supporting these results, we recently observed menopausal transition to associate with increased blood triglyceride and LDL-cholesterol levels, although we also observed HDL-cholesterol levels to rise (58). The role of HDL-cholesterol, as such, in CV protection is questionable. Ding and Manson (2021) suggested that despite of the observed associations between higher HDL-cholesterol with lower carotid intima-media thickness among postmenopausal women, the HDL particle number and size – indicators of the functionality of HDL – may be more potent measures of CV health (59). In the current study, total and LDL-cholesterol levels were higher in menopausal women compared to women of reproductive age, but no significant difference was observed in triglycerides or HDL-cholesterol. However, since women of reproductive age also had healthier body composition, higher absolute and relative V̇O_2PEAK_, lower blood pressure and less stiff arteries than menopausal women, they can be considered to be cardiometabolically healthier.

### Limitations

This study combined data from two separate studies, MEndEx and EsmiRs, in a secondary analysis to investigate associations between sex hormone levels and arterial stiffness across a wide age-range, including both women with natural hormonal status and women using external hormonal medication for contraception or menopausal symptoms. As neither of the studies was originally designed for this particular study question, we did not have *a priori* power calculations done before the onset of the study, and thus, our study may have remained underpowered. However, we did have 26 women in the REPRO and 39 women in the MENO group, which is similar and, in some cases, higher to the group sizes used in previous studies. Furthermore, our study contains multiple measurement points (4 for the REPRO and 2 for the MENO groups), which allowed us to use GEE-models with a maximum of 172 and a minimum of 159 data points across the whole study (wide age-range models) and with 36 to 75 data points across the age-group focused analyses. Nevertheless, specifically age-group focused analyses had a rather small sample size, the smallest being *n* = 5 for MENO-PERI and *n* = 8 for MENO-HT groups, which may have limited the statistical power of the study. The MEndEx and EsmiRs studies used a free-living live sample of women, and therefore, the use of COC and HT were not controlled in terms of length and used product, which may also have caused variation to our results. All used, however, combined specimens with estrogenic and progestogenic effective agents. It is also a limitation that our study lacked quantification of exogenous hormones, particularly in COC- and HT-users; thus, our study was limited to rely on analysis of endogenous hormones. In addition, although we attempted to carefully monitor the menstrual cycle by counting days from the commencement of bleeding and by urine tests to identify ovulation, the considerable variation in systemic hormones within each menstrual cycle phase, particularly in LH within the ovulation phase, indicates that we did not completely succeed. On the other hand, we were able to reduce some technical variation between the MEndEx and EsmiRs studies as both were performed at the same research environment by using the same measurement protocol and arteriography-device to assess arterial stiffness, which ensures that no device-based systematic bias took place. Some clear deviations remained, including the younger cohort being physically fitter than the older cohort. This difference was controlled by including relative V̇O_2PEAK_ and fat mass as covariates in the analysis. Taken together, although we did have a longitudinal design, our study should be considered as explorative rather than definitive, and the results should be interpreted with caution keeping these limitations in mind.

### Potential Cardiovascular Significance of the Results

Our female population is considered to be a healthy study sample without major CV risk factors such as obesity, smoking, and diagnosis of or treatment for hypertension, diabetes or dyslipidaemia. Nevertheless, at the group mean level, the observed menopausal women’s PWV 9.1 m/s was slightly higher than the common reference value 8.3 m/s provided for age category 50–59 years (60). The observed younger women’s (age-range: 19–37 years) group mean level 6.4 m/s was in line with the reference values 6.2 and 6.5 m/s available for age categories <30 years and 30–39 years, respectively (60). We were unable to find sex- and device-specific reference values for PWV and AIx covering the age-range of our study. Paiva et al. (2020) (13) provides reference values for 30–39 years old women to be 5.0–6.1 m/s for PWV and 12–34% for Aix and corresponding values for 50–59 years old women to be 7.0–8.3 m/s and 7–39% by using Mobil-O-Graph PWV -device, which is a similar oscillometric cuff-based device than the Arteriograph used in the current study. However, different devices are likely to deviate in the absolute values they provide (61, 62). Taken that comparing exact measurement values between devices is not recommendable and the lack of proper normal values specifically for Arteriograph, we cannot conclude if the observed PWV 9.1 m/s and AIx 46% among menopausal women are merely a reflection of healthy vascular aging or if they could be interpreted as early signs of worsening CV health. However, some indication of the clinical significance can be obtained by inspecting the magnitude of group differences obtained by the same device. In our study sample, the reproductive-age about 25-years-old women had 2.7 m/s lower PWVao than the menopausal-age about 55-years-old women. The mean difference in AIx% (29.4%) was also in favor of the younger women. Based on the study by Vlachopaoulos et al., 2010 (7), 1 m/s increase in PWV corresponds to over 10% higher risk of cardiovascular events. Thus, the observed age-group difference can be considered clinically significant. Since menopausal transition is a known period of detrimental changes in several CV risk factors in addition to vascular aging and stiffening (63), healthcare providers need to be alert with menopausal women to offer early lifestyle intervention recommendations before risk factors accumulate and, in the worst scenario, lead to the onset of CV disease.

### Conclusions

In conclusion, age-related differences in arterial stiffness are not explained by sex hormones over the effect of aging itself. Menstrual and COC-pill cycles are differentially associated with arterial stiffness, indicating that sex hormone cycling may affect arterial elasticity during the reproductive period of women’s life. After menopause, HT use does not seem to add-back the potential beneficial effects of cyclic systemic hormones, but may, in contrast, associate with higher arterial stiffness compared to postmenopausal non-HT-users. More research is warranted to resolve potential sex hormone-mediated mechanisms affecting arterial elasticity and to understand which of the endogenous and exogenous hormones have functional or biomarker relevance to arterial health.

## Data Availability Statement

The original contributions presented in the study are included in the article/**Supplementary Material**. Further inquiries can be directed to the corresponding author.

## Ethics Statement

The studies involving human participants were reviewed and approved by the Ethical Committee of the University of Jyväskylä and the Ethics committee of the Central Finland Health Care District. The patients/participants provided their written informed consent to participate in this study.

## Author Contributions

EKL, JEK, UK, EAH, PA, JAL, and JKI designed the study. Data were collected by JEK, EL, EP, H-KJ, JAL and JKI. Analysis was carried out and the initial manuscript was drafted by EKL, JEK, and SL. EKL and JKI take final responsibility for this article. All authors provided contributions to study conception and design, acquisition of data or analysis and interpretation of data, drafting the article or revising it critically for important intellectual content, and final approval of the version to be published.

## Funding

This study was supported by the Academy of Finland grants 309504, 314181, 335249, and 330281 to EKL, by the Finnish Foundation for Cardiovascular Research to EAH, and by Urheiluopistosäätiö grant 20190110 to JKI. The funders had no role in the study design, data collection and analysis, decision to publish or preparation of the manuscript.

## Conflict of Interest

The authors declare that the research was conducted in the absence of any commercial or financial relationships that could be construed as a potential conflict of interest.

## Publisher’s Note

All claims expressed in this article are solely those of the authors and do not necessarily represent those of their affiliated organizations, or those of the publisher, the editors and the reviewers. Any product that may be evaluated in this article, or claim that may be made by its manufacturer, is not guaranteed or endorsed by the publisher.
