# Supplementary material for: Associations of Sex Hormones and Hormonal Status With Arterial Stiffness in a Female Sample From Reproductive Years to Menopause
Source: Front Endocrinol (Lausanne). 2021 Nov 30;12:765916. doi: 10.3389/fendo.2021.765916 (PMC8669797; doi:10.3389/fendo.2021.765916)
Supplement: Supplementary file 1 [file Table_1.docx]

# Supplementary Material

**TABLE S1.** Parameters of cardiovascular health within sub-groups.

**Supplemental table S1.** Parameters of cardiovascular health within sub-groups.

|  | REPRO group | | |  | MENO group | | | |
| --- | --- | --- | --- | --- | --- | --- | --- | --- |
|  | -NAT  *n* =16 | -COC  *n* =10 | *P* -value |  | -PERI  *n* =5 | -POST  *n* =26 | -HT  *n* =8 | *P*-value |
| **Blood lipids and lipoproteins^a^** |  |  |  |  |  |  |  |  |
| Triglycerides (mmol/l) | 0.7 ± 0.1 | 1.0 ± 0.3 | **0.006** |  | 0.8 ± 0.2 | 1.0 ± 0.4 | 1.0 ± 0.2 | 0.378 |
| Total cholesterol (mmol/l) | 4.3 ± 0.6 | 5.0 ± 1.0 | 0.177**^c^** |  | 5.2 ± 0.5 | 6.1 ± 1.2 | 5.4 ± 0.6 | 0.094^d^ |
| LDL-cholesterol (mmol/l) | 2.2 ± 0.4 | 2.6 ± 0.9 | 0.428**^c^** |  | 2.9 ± 0.6 | 3.7 ± 110 | 3.1 ± 0.6 | 0.184^d^ |
| HDL-cholesterol (mmol/l) | 1.8 ± 0.3 | 1.9 ± 0.4 | 0.337 |  | 2.1 ± 0.5 | 2.1 ± 0.5 | 1.8 ± 0.5 | 0.440 |
| **Body composition** | | | | | | | | |
| Body mass index (kg/m^2^) | 24.0 ± 2.5 | 21.2 ± 1.6 | **0.005** |  | 25.2 ± 3.4 | 24.4 ± 2.3 | 24.5 ± 3.2 | 0.814 |
| Fat free mass (kg) | 52.1 ± 5.6 | 50.8 ± 6.5 | 0.580 |  | 49.1 ± 4.4 | 46.1 ± 4.8 | 45.7 ± 4.2 | 0.389 |
| Fat mass (kg) | 14.6 ± 5.5 | 11.4 ± 1.9 | 0.085 |  | 22.2 ± 5.6 | 20.8 ± 5.1 | 22.1 ± 6.5 | 0.755 |
| **Cardiorespiratory fitness^b^** | | | | | | | | |
| V̇O_2PEAK_ (l/min) | 3.0 ± 0.4 | 2.8 ± 0.4 | 0.333 |  | 2.3 ± 0.3 | 2.2 ± 0.4 | 1.9 ± 0.4 | 0.105 |
| Relative VO_2PEAK_ (ml/kg FFM/min) | 58.6 ± 11.4 | 54.4 ± 4.5 | 0.330**^c^** |  | 46.0 ± 4.0 | 47.3 ± 6.1 | 41.3 ± 6.5 | 0.101^d^ |
| Resting heart rate (bpm) | 56.0 ± 10.9 | 61.7 ± 11.2 | 0.286^c^ |  | 53.0 ± 3.7 | 57.4 ± 9.7 | 59.4 ± 4.8 | 0.127^d^ |
| **Blood pressure and arterial stiffness** | | | | | | | | |
| Systolic blood pressure (mmHg) | 108.1 ± 6.6 | 117.8 ± 9.8 | **0.017^c^** |  | 133.6 ± 19.5 | 122.5 ± 12.9 | 122.9 ± 18.8 | 0.448^c^ |
| Diastolic blood pressure (mmHg) | 59.7 ± 5.6 | 68.4 ± 9.9 | **0.008** |  | 74.6 ± 5.3 | 73.1 ± 7.5 | 70.3 ± 9.5 | 0.565 |
| Mean arterial pressure (mmHg) | 75.8 ± 5.3 | 84.9 ± 10.0 | **0.021** |  | 94.3 ± 9.0 | 89.6 ± 9.0 | 87.8 ± 12.4 | 0.504 |
| Aortic pulse wave velocity (m/s) | 6.2 ± 0.7 | 6.9 ± 0.7 | **0.036^c^** |  | 9.5 ± 1.7 | 8.6 ± 1.9 | 10.3 ± 1.9 | 0.072^d^ |
| Augmentation index (%) | 16.3 ± 5.4 | 16.7 ± 6.0 | 1.0 |  | 47.8 ± 14.8 | 44.7 ± 7.9 | 46.3 ± 13.5 | 0.347^d^ |

Values are mean ± standard deviation. V̇O_2PEAK_=peak oxygen uptake, FFM=fat free mass, LDL=low-density lipoprotein, HDL=high-density lipoprotein.Missing data: ^a^REPRO group: *n* = 1, ^b^REPRO-NAT group: *n* = 2 and REPRO-COC group: *n* = 2. ^c^Mann-Whitney U test was used for statistical testing. ^d^Kruskal-Wallis test was used for statistical testing. Significant *P*-values are bolded.
